# Combining omics data to identify genes associated with allergic rhinitis

**DOI:** 10.1186/s13148-017-0310-1

**Published:** 2017-01-18

**Authors:** Andréanne Morin, Michel Laviolette, Tomi Pastinen, Louis-Philippe Boulet, Catherine Laprise

**Affiliations:** 1 Department of Human Genetics, McGill University and Genome Quebec Innovation Centre, 740 Dr. Penfield Avenue, Montréal, Québec H3A 1A5 Canada; 2 Département des sciences fondamentales, Université du Québec à Chicoutimi, 555 boulevard de l’Université, Saguenay, Québec G7H 2B1 Canada; 3 Institut Universitaire de Cardiologie et de Pneumologie de Québec, Université Laval, 2725 chemin Sainte-Foy, Québec, Québec G1V 4G5 Canada

**Keywords:** Allergic rhinitis, Asthma, GWAS, EWAS, mQTLs, Omics

## Abstract

Allergic rhinitis is a common chronic disorder characterized by immunoglobulin E-mediated inflammation. To identify new genes associated with this trait, we performed genome- and epigenome-wide association studies and linked marginally significant CpGs located in genes or its promoter and SNPs located 1 Mb from the CpGs, by identifying *cis* methylation quantitative trait loci (mQTL). This approach relies on functional cellular aspects rather than stringent statistical correction. We were able to identify one gene with significant *cis*-mQTL for allergic rhinitis, caudal-type homeobox 1 (*CDX1*). We also identified 11 genes with marginally significant *cis*-mQTLs (*p* < 0.05) including one with both allergic rhinitis with or without asthma (*RNF39*). Moreover, most SNPs identified were not located closest to the gene they were linked to through *cis*-mQTLs counting the one linked to *CDX1* located in a gene previously associated with asthma and atopic dermatitis. By combining omics data, we were able to identify new genes associated with allergic rhinitis and better assess the genes linked to associated SNPs.

## Introduction

Allergic rhinitis is one of the most common allergies worldwide and one of the most common chronic disorders among children and adults [1]. Early sensitization to aeroallergens and food combined with the presence of atopic dermatitis, characterized by an immunoglobulin E (IgE)-mediated inflammation, can result in the development of asthma and/or allergic rhinitis later in life in a process called “atopic march” [2]. Genetic studies identified hundreds of genes associated with allergic rhinitis, and genome-wide association studies (GWASs) pinpointed single nucleotide polymorphisms (SNPs) associated with its development [3, 4]. However, a majority of identified SNPs lie in the non-coding genomic region, making it difficult to identify the targeted genes. Given that DNA methylation may have an impact on gene regulation [5], the probability of detecting true positive associations should be improved by combining nominally significant data from genomics and epigenomics and linking them by quantitative trait loci (QTL) analysis. Methylation QTLs (mQTLs) allow assessing the impact of DNA-sequenced variations (SNPs) on DNA methylation. They have been assessed in different tissues and cell types and were shown to overlap with GWAS hits [6–9]. We used this approach to identify allergic rhinitis genes and illustrate its usefulness in the context of a complex trait.

## Materials and methods

### Individual selection, characterization, and sample preparation

We used data available from the Saguenay–Lac-Saint-Jean (SLSJ) asthma familial collection from Québec, Canada, that has data for rhinitis and allergies (Table 1). This population is known for its founder effect and is more homogeneous than a cosmopolitan population [10, 11]. Individuals affected with rhinitis and allergies, with or without asthma, were analyzed as cases. Individuals with no rhinitis, allergies, and asthma were considered as controls. In this study, patients were defined as asthmatics based on if they either had a reported history of asthma (validated by a physician) or if at recruitment they manifested asthma-related symptoms and positive PC_20_ (<8 mg/ml) [12]. Rhinitis was self-reported, and the subject had to answer “yes” to at least one of the following questions: Have you ever had rhinitis?; Have you ever had hay fever?; and Have you ever had sneeze or rheum after contact with the following: hay, flowers, animals, and dust? Allergy was defined by a skin prick test for 26 aeroallergens (≥3 mm). All subjects were recruited and evaluated out of the pollen season [12]. Recruitment and clinical evaluation of individuals as well as phenotype description can be found in Laprise [12]. All subjects gave their informed consent, and the project was approved by the research ethic committee of the Centre intégré universitaire de santé et de services sociaux du SLSJ.

**Table 1:** General characterization of individuals analyzed in the study

	GWAS samples			EWAS samples		
	Controls^a^	Allergic rhinitis^b^	Allergic rhinitis combined with asthma^c^	Controls^a^	Allergic rhinitis^b^	Allergic rhinitis combined with asthma^c^
Number of samples	187	125	321	31	30	48
M/F ratio	1:1.13	1:0.87	1:0.87	1:1.60	1:0.88	1:0.78
Age, mean (range)^d^	43 (3–85)	37 (5–93)	28 (5–83)	29 (1–53)	28 (1–59)	28 (5–55)
Age median^d^	41	38	26	35	30	26
Smoking status, *n* (%)^e^						
Non-smoker	82 (44)	64 (51)	219 (68)	14 (45)	18 (60)	36 (75)
Ex smoker	61 (33)	37 (30)	53 (17)	8 (26)	6 (20)	4 (8)
Smoker	43 (23)	21 (17)	44 (14)	9 (29)	5 (17)	7 (15)
IgE, μg/L (SD)^f^	202.85 (1373.66)	411.27 (852.17)	856.45 (2075.62)	67.10 (90.45)	575.40 (1380.45)	597.73 (242.50)

^a^Defined as not affected by either asthma, allergies, or rhinitis. ^b^Defined as being affected with both allergy and rhinitis. Allergic rhinitis phenotype is available for all samples. Allergy is defined as at least one positive response on skin prick testing (wheal diameter ≥3 mm at 10 min). Rhinitis is self-reported, and the subject had to answer “yes” to at least one of the following questions: Have you ever had rhinitis?; Have you ever had hay fever?; and Have you ever had sneeze or rheum after contact with hay, flowers, animals, and dust? Can be either combined^c^ or not^b^ with asthma. ^d^Age difference between groups were assessed using an unpaired *t* test. GWAS: controls vs allergic rhinitis *p* = 0.078 and control vs allergic rhinitis combined with asthma *p* = 1.2e−15. EWAS: controls vs allergic rhinitis *p* = 0.078 and control vs allergic rhinitis combined with asthma *p* = 0.43. ^e^Smoking status available for 186 controls, 122 allergic rhinitis, and 316 allergic rhinitis combined with asthma subjects for genome-wide association study (GWAS) samples and 31 controls, 29 allergic rhinitis, and 47 allergic rhinitis combined with asthma subjects for epigenome-wide association study (EWAS) samples. Differences between groups were assessed using a chi-square test. GWAS: controls vs allergic rhinitis *p* = 0.0045 and control vs allergic rhinitis combined with asthma *p* = 1.25e−19. EWAS: controls vs allergic rhinitis *p* = 0.049 and control vs allergic rhinitis combined with asthma *p* = 7.7e−3. ^f^Geometric mean and standard deviation (SD) for the immunoglobulin E (IgE) serum concentration calculated for 175 controls, 116 allergic rhinitis, and 302 allergic rhinitis combined with asthma subjects for GWAS samples and all subjects for EWAS samples. IgE level differences between groups were assessed using an unpaired *t* test. GWAS: controls vs allergic rhinitis *p* = 0.145 and control vs allergic rhinitis combined with asthma *p* = 2.2e−3. EWAS: controls vs allergic rhinitis *p* = 0.003 and control vs allergic rhinitis combined with asthma *p* = 0.90. Sex, age, cell count, and smoking status were used as covariates in the analyses

### Genome-wide association study

A total of 508 subjects (321 cases and 187 controls) and 312 subjects (125 cases and 187 controls) were included in the analysis for allergic rhinitis with or without asthma, respectively. The same group of controls was used to compare to both phenotypes (i.e., allergic rhinitis and allergic rhinitis with asthma). DNA extraction, genotyping methods, and statistical analyses were described previously [12]. Genotyping was performed using the Illumina 610K Quad array (Illumina, San Diego, CA, USA). Association test was performed using a quasi-likelihood score test using the MQLS program (Release 1.5, http://www.stat.uchicago.edu/~mcpeek/software/MQLS/index.html), which allows performing case-control association analysis using related individuals [13]. The kinship coefficient was calculated using KinlnbCoef program (version 1.1, http://www.stat.uchicago.edu/~mcpeek/software/KinInbcoef/index.html). We included in the analysis SNPs with minor allele frequency (MAF) >0.05, *p* value for Hardy-Weinberg equilibrium >0.0001, and overall call rate >95%. Samples with genotyping rate <95% were excluded. A total of 633 samples (321 subjects with allergic rhinitis with asthma, 125 subject with allergic rhinitis only, and 187 controls (used to compare to both phenotypes)) and 506,388 SNPs were included in the analysis.

### Epigenome-wide association study

A total of 31 controls and 48 cases for allergic rhinitis with asthma or 30 cases for allergic rhinitis alone were included in the epigenome-wide association study (EWAS) analysis. These samples are a subset of the ones used in the GWAS analysis. Unrelated subjects were included based on having allergic rhinitis with or without asthma and having no asthma, allergies, or rhinitis and based on having high or low levels of IgE. DNA extraction and sodium bisulfite conversion methods were described previously [14]. The assay was carried out on the Infinium HumanMethylation450 BeadChip array (Illumina, San Diego, CA, USA). The analysis was performed using the RnBeads Bioconductor R package [15]. We removed probes with at least one of the following characteristics: (1) weak signal (*p* > 0.01) (2128 CpG sites), (2) SNP-enriched sites (4100 sites), (3) out of a CpG context (not on a CG) (3149 sites), or (4) located on sex chromosomes (11,129 sites). A total of 465,071 CpG sites were analyzed initially. Signal was then normalized, first by scaling to the internal controls using the methylumi R package [16], then by applying the method of subset-quantile within array normalization (SWAN) implemented in the minfi R package [17, 18]. A total of 2203 sites were removed due to missing data. We removed probes that mapped multiple genomic regions (≥90% sequence similarity), that have a variant less than 10 bp from the CpG, or that have ≥2 SNPs in it. A total of 374,498 CpG sites (80.5%) were analyzed for differential DNA methylation using limma R package [19]. All samples had cell counts for eosinophils, basophils, monocytes, lymphocytes, and neutrophils. The cell percentages were used as covariates as well as sex, age, smoking status, and batch effect.

### Methylation quantitative trait loci analysis

To perform the mQTL analyses, we used associated SNPs (*p* < 0.05) and CpGs (*p* < 0.05 and Δ*β* > 0.05) in the GWAS and EWAS for both traits. We kept associated CpGs that were located in either the gene body or 1.5-kb upstream of the transcription start site, keeping 88 and 144 CpGs for allergic rhinitis with or without asthma, respectively. SNPs were kept if present in all samples and if the three genotype groups (homozygous reference, heterozygous and homozygous alternative) were observed at least five times. A total of 529 and 625 SNPs were included in the analysis for allergic rhinitis with or without asthma, respectively. We analyzed *cis*-mQTLs where the CpG-SNP combination was less than 1 Mb apart from each other based on the distance used by the GTEX consortia for their *cis* expression quantitative trait loci (*cis-*eQTLs) (http://www.gtexportal.org/home/documentationPage). We used a Bonferroni correction to evaluate significance thresholds. We computed mQTLs for these SNP-CpG pairs using an additive linear model using the R package MatrixEQTL [20]. Same covariates as in EWAS were included in this analysis. A total of 274 (Bonferroni *p* = 0.05/274 = 1.8e−4) and 500 (Bonferroni *p* = 0.05/500 = 1e−4) CpG-SNP comparisons were performed for allergic rhinitis with or without asthma, respectively.

## Results and discussion

**Fig. 1:**
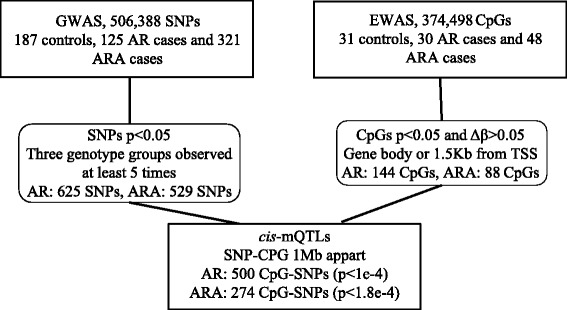
Flowchart presenting our approach combining genome-wide association study (GWAS) and epigenome-wide association study (EWAS) hits to identify *cis* methylation quantitative trait loci (mQTLs) that could be associated with allergic rhinitis with (ARA) or without asthma (AR). We first performed GWAS and EWAS separately for AR and ARA. We then selected marginally associated SNPs (*p* < 0.05) where the three genotyping groups were observed at least five times. We also selected marginally associated CpGs (*p* < 0.05) that had a Δ*β* > 0.05 and that were located in the gene body or 1.5-kb from the transcription start site (TSS). We then linked the SNPs and CpGs that were 1 Mb apart by performing *cis-*mQTLs for both AR and ARA. We used Bonferroni *p* value cutoffs to assess significance

In this study, we used a novel approach that links genetic (SNPs) and functional (CpGs) data through the use of mQTLs identifying new genes associated with allergic rhinitis with or without asthma (Fig. 1). It relies on functional cellular data and reduces the stringent cutoff normally used in GWAS. Even though this is a pilot experiment with small number of samples, we identified one significant *cis-*mQTL for allergic rhinitis located in caudal-type homeobox 1 (*CDX1*) (*p* = 6.41e−5) (Table 2). We also observed nine nominally associated *cis-*mQTLs located in five genes for allergic rhinitis and 16 located in nine genes for allergic rhinitis with asthma (Table 2). One gene was reported being associated in both traits: ring finger protein 39 (*RNF39*). It has the highest number of mQTLs identified in both allergic rhinitis with (four) or without asthma (five).

**Table 2:** Genes with *cis*-mQTL sites significantly associated with allergic rhinitis with or without asthma

Trait	Gene	Locus	mQTLs	GWAS analysis		EWAS analysis		
			*p* value	SNP	*p* value	CpGs	Δ*β* ^a^	*p* value
Allergic rhinitis	***CDX1*** ^***b***^	chr5q32	**6.41e−5**	rs888989	**0.0038**	cg18424208	−5.19	**0.0002**
	*PPAN-P2RY11*	chr19p13.2	0.0245	rs3752199	0.0346	cg24118856	7.51	4.39e−5
	*RNF39* ^*c*^	chr6p22.1	0.0090	rs2844833	0.0270	cg05563515	10.11	0.0212
			0.0229	rs2844833	0.0270	cg24637044	5.85	0.0132
			0.0265	rs2844833	0.0270	cg01286685	7.78	0.0266
			0.0411	rs2523872	0.0123	cg10930308	9.50	0.0255
			0.0499	rs2523872	0.0123	cg01286685	7.78	0.0266
	*SRRT*	chr7q22.1	0.0412	rs6942824	0.0224	cg10426581	5.26	0.0096
Allergic rhinitis with asthma	*ADORA1*	chr1q32.1	0.0337	rs6661284	0.0337	cg19315653	−6.26	0.0315
	*ITGB2*	chr21q22.3	0.0381	rs7275203	0.0381	cg18012089	6.10	0.0068
	*LINC00336*	chr6p21.31	0.0073	rs9461924	0.0073	cg04329454	−7.16	0.0015
	*MFSD6L*	chr17p13.1	0.0120	rs9895992	0.0120	cg11685316	5.01	0.0072
	*PCDH8*	chr13q14.3	0.0152	rs732774	0.0295	cg14950829	7.53	0.0097
			0.0135	rs3742297	0.0480	cg14950829	7.53	0.0097
			0.0259	rs1801249	0.0296	cg14950829	7.53	0.0097
			0.0259	rs4943046	0.0298	cg14950829	7.53	0.0097
	*PITX2*	chr4q25	0.0257	rs2067004	0.0272	cg13385016	5.06	0.0240
			0.0249	rs9992755	0.0289	cg13385016	5.06	0.0240
	*RNF180*	chr5q12.3	0.0130	rs7713289	0.0130	cg17370163	5.43	0.0021
	*RNF39* ^*c*^	chr6p22.1	0.0133	rs2517504	0.0047	cg03343571	9.19	0.0451
			0.0171	rs2517504	0.0047	cg01286685	8.21	0.0478
			0.0401	rs2535238	0.0248	cg01286685	8.21	0.0478
			0.0499	rs2523872	0.0299	cg01286685	8.21	0.0478
	*ZFPM1*	chr16q24.2	0.0304	rs750740	0.0304	cg04983687	5.53	0.0056

^a^Δ*β* and *p* values for CpG sites and SNPs forming a *cis*-mQTL. A negative Δ*β* indicates a decrease in the percentage of methylation for cases compared to controls. All loci refer to the human hg19 reference genome

^b^CDX1 is the only gene for which the mQTL *p* value survives multiple correction (*p* < 1e-4)

^c^ *RNF39* is the only gene marginally associated in both traits

The significantly or nominally associated genes were not associated with any related trait before. Interestingly, the majority of the genes linked to a SNP by the *cis*-mQTLs are not the closest ones, thus would not be the ones reported in a regular GWAS study. For example, all of the significant SNPs reported for the *RNF39 cis*-mQTLs are located 300 kb to 1 Mb away from the gene and are located closer to other genes, which were previously associated with pulmonary function (rs2844833-*HLA-F* [21], rs2523872-*MUC22* [21], rs2517504-*HCG22* [21, 22], rs2535238-*ZFP57* [21]). The best example remains the one for the significantly associated mQTL that links rs888989 to a CpG located in the promoter region of the *CDX1* gene*.* The SNP is located in an intron of TNFAIP3 interacting protein 1 (*TNIP1*) and 900 kb from *CDX1*. The former was previously associated with atopic dermatitis [23] and asthma [24]. According to the GTEx Portal (http://www.gtexportal.org/), rs888989 and *CDX1* form an expression quantitative trait loci (eQTL) in the lungs (*p* = 0.04), which is not the case for *TNIP1* (*p* = 0.94). This reinforces the possible implication of this gene in allergic rhinitis and shows that our method may better assess the true genes of interest linked to the associated SNPs.

The originality of our approach resides in combining GWAS and EWAS nominally associated SNPs and CpGs, using *cis*-mQTL data, to identify genes of interest in this disease. This method has the potential to reduce false negative findings by relying on the cellular mechanisms of gene regulation compared to the use of stringent statistical corrections. The use of a well-described collection coming from a founder population and including subjects selected based on the same precise criteria allowed a more unified genetic background and phenotype. However, since this is a pilot study, the limited number of samples included in the EWAS and the GWAS may constrain the power of the findings. We were not able to test SNPs previously associated with the trait in previous GWASs because they did not meet the criteria to be included in the mQTL analysis. We also analyzed SNPs and CpGs preselected in the arrays by the manufacturers, thus excluding potentially important SNPs or CpG sites, which are not in linkage disequilibrium. DNA methylation analysis using whole blood could have limited the findings, even if correction for cell counts was included in our model. Apart from the limitations, we showed that our approach is promising and acknowledging for the lack of power in future studies will permit to better pinpoint genes of interests for different traits. Studying other tissues implicated in allergic rhinitis trait, like nasal or lung cells, could also reveal other genes implicated in the physiopathology. Genes identified in this study, notably *CDX1*, are worthwhile to be further investigated to understand the allergic rhinitis pathogenesis and the atopic march.

## Abbreviations

AR: Allergic rhinitis
ARA: Allergic rhinitis with asthma
CDX1: Caudal-type homeobox 1
eQTL: Expression quantitative trait loci
EWAS: Epigenome-wide association study
GWAS: Genome-wide association study
HCG22: HLA complex group 22
HLA-F: Major histocompatibility complex, class I, F
IgE: Immunoglobulin E
MAF: Minor allele frequency
mQTL: Methylation quantitative trait loci
MUC22: Mucin 22
RNF39: Ring finger protein 39
SLSJ: Saguenay–Lac-Saint-Jean
SNP: Single nucleotide polymorphism
SWAN: Subset-quantile within array normalization
TNIP1: TNFAIP3 interacting protein 1
TSS: Transcription start site
ZFP57: Zinc finger protein 57
